# Target coverage of daily cone-beam computed tomography in breath-hold image-guided radiotherapy for gastric lymphoma

**DOI:** 10.1259/bjro.20200062

**Published:** 2021-07-05

**Authors:** Shigeo Takahashi, Takamasa Nishide, Masato Tsuzuki, Hiroki Katayama, Masahide Anada, Toshifumi Kinoshita, Shohei Kozai, Toru Shibata

**Affiliations:** 1 Department of Radiation Oncology, Kagawa University Hospital, Kagawa, Japan; 2 Department of Clinical Radiology, Kagawa University Hospital, Kagawa, Japan

## Abstract

**Objectives:**

We evaluated retrospectively the daily target coverage using cone-beam computed tomography (CBCT) in breath-hold image-guided radiotherapy (BH-IGRT) for gastric lymphoma.

**Methods:**

BH-IGRT was performed using a prescribed dose of 30.6 Gy in 17 fractions for the whole stomach. We assessed the target coverage of the whole stomach on daily CBCT images [daily clinical target volume (CTV)], which was delineated individually by two observers. We evaluated V_95%_ (percentage of volume receiving ≥95% of the prescribed dose) of daily CTV.

**Results:**

In total, 102 fractions from 6 patients were assessed. The mean V_95%_ of daily CTV was 97.2%, which was over 95%. In two of six patients, the V_95%_ of daily CTV was over 95% for either observer in all fractions. One patient had significant interobserver variation (*p* = 0.013). In 95 fractions (93%), the V_95%_ of daily CTV was over 95% for either observer.

**Conclusion:**

Daily target coverage for CTV in BH-IGRT for gastric lymphoma seems to be favorable, even when using CBCT.

**Advances in knowledge:**

A previous study ascertained good daily target coverage in BH-IGRT for gastric lymphoma using in-room CT. Even when using CBCT in our study, daily target coverage for CTV in BH-IGRT for gastric lymphoma seems to be favorable.

## Introduction

The respiratory motion of abdominal tumors is an obstacle to radiotherapy (RT).^1^ To assess the respiratory motion, four-dimensional computed tomography (4DCT) is utilized for radiation treatment planning in gastric lymphoma.^2,3^ Recently, breath-hold image-guided radiotherapy (BH-IGRT) has been applied to treat gastric lymphoma to overcome the respiratory motion and reduce radiation doses for normal tissues.^4,5^ It is important to assess whether the target is irradiated properly but data on daily target coverage of radiation is limited in BH-IGRT for gastric lymphoma. A previous study, using in-room computed tomography (CT), reported good daily target coverage.^6^ Data regarding daily target coverage using cone-beam CT (CBCT) are needed as in-room CT is not available in every institution; in contrast CBCT is widely used.^7,8^ Therefore, we studied the daily target coverage for gastric lymphoma using CBCT in BH-IGRT.

## Methods and materials

### Inclusion criteria

This retrospective study was approved by the institutional ethics committee (number 2019-064). The inclusion criteria for this study were as follows: (1) patients with gastric lymphoma; and (2) BH-IGRT was performed using 30.6 Gy in 17 fractions for the whole stomach between 2016 and 2019 in our institution (Kagawa University Hospital).

### Radiation treatment planning

**Figure 1.**
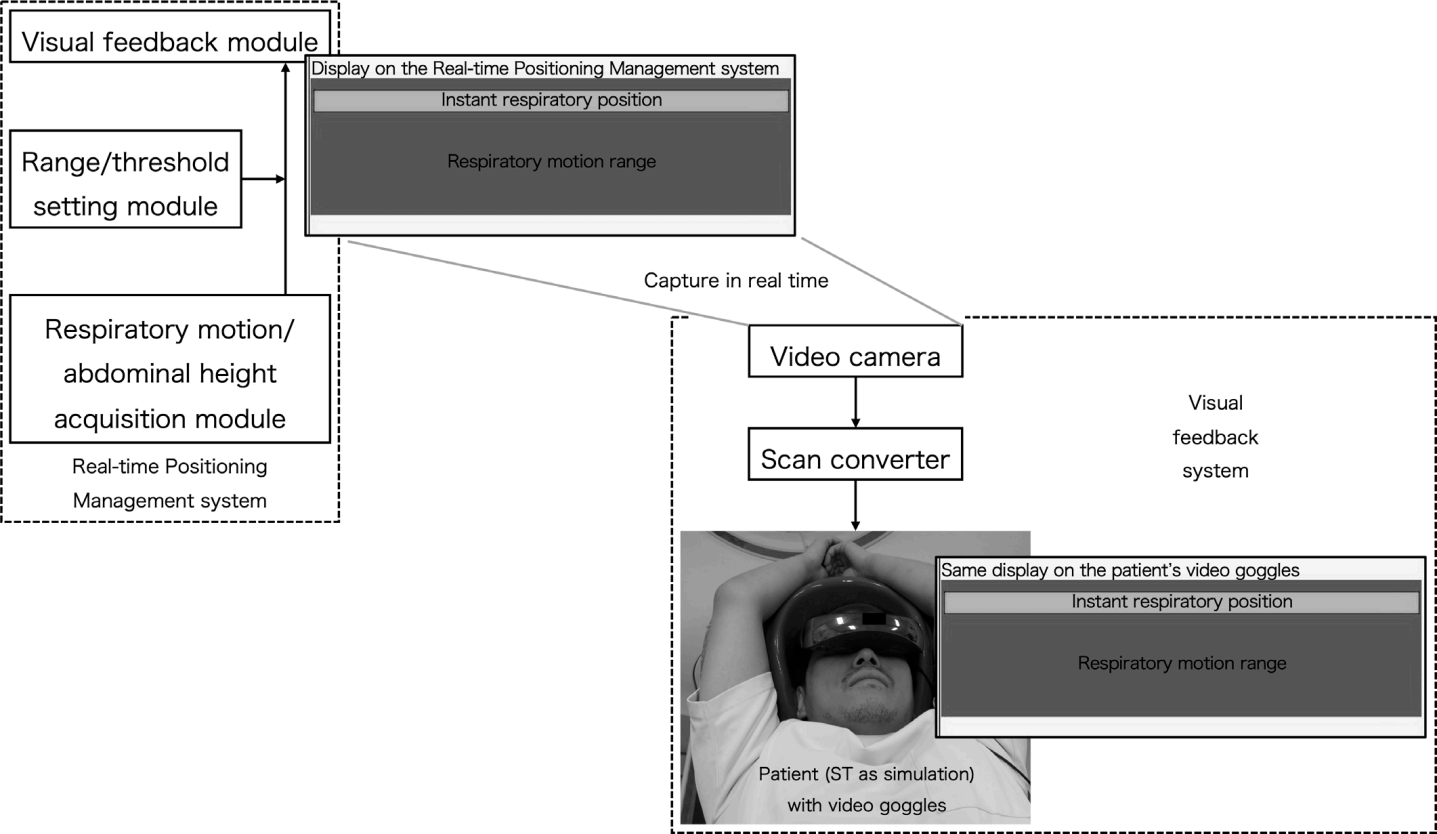
Schema of our visual feedback system. Through capturing the display on the Real-time Positioning Management system with a video camera in real time, the same display can be seen on the patient’s video goggles. Using the bar of instant respiratory position, we can instruct the patient in the timing of the breath-hold.

Patients abstained from one meal before planning CT. Premedication was not used for planning CT. Patients were fixed using the ESFORM immobilization system (Engineering System, Nagano, Japan). As reported previously, in studies of BH-IGRT,^9,10^ we used a visual feedback method for breath-hold (BH) with the Real-time Positioning Management system (Varian Medical Systems, Palo Alto, CA) and video goggles. The schema of our visual feedback system is shown in Figure 1. We obtained three sets of breath-hold computed tomography (BH-CT) images to confirm the reproducibility of BH for each patient. We delineated the whole stomach on the three sets of BH-CT images. The three delineated whole stomach was defined as the clinical target volume (CTV). The planning target volume (PTV) was made by CTV plus a 0.8 cm margin. The dose prescription at the isocenter was 30.6 Gy in 17 fractions using 4–5 non-coplanar 10 MV photon beams from a linear accelerator (Clinac iX; Varian Medical Systems, Palo Alto, CA).

### Daily treatment

**Figure 2.**
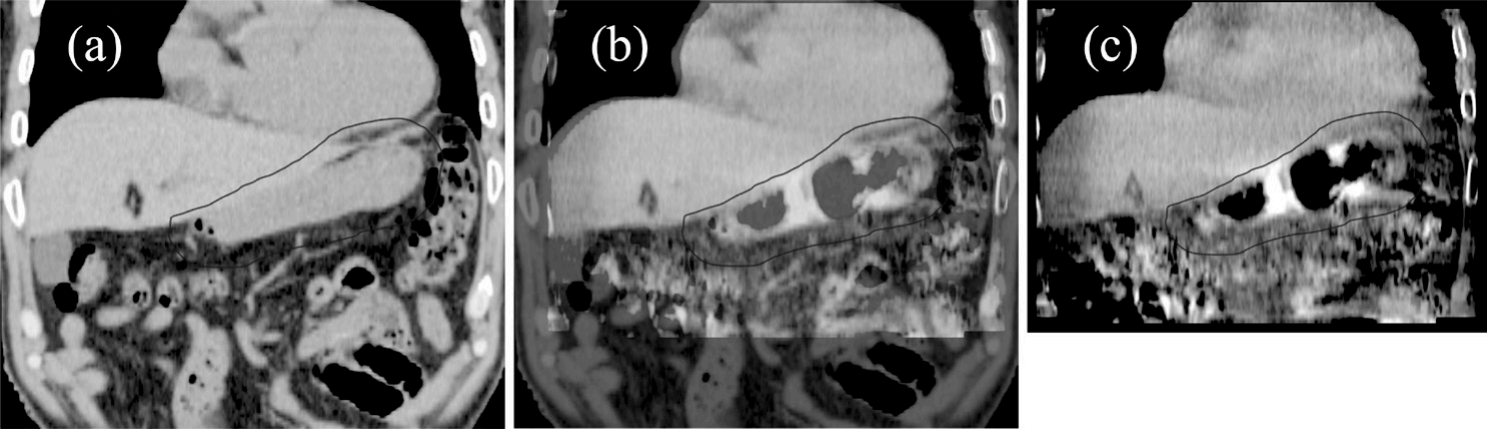
Example of the position matching: (**a**) Planning breath-hold computed tomography image; (**b**) Blended image of (**a**) and (**c**); (**c**) Daily BH-CBCT image. Using the daily BH-CBCT image, radiation oncologists and radiological technologists matched the position of the daily whole stomach within the planning target volume (black bold line). BH-CBCT, breath-hold cone-beam computed tomography.

Patients abstained from one meal before daily BH-IGRT. Premedication was not used for daily BH-IGRT. After manual setup using a laser, two orthogonal kilovoltage (kV) radiographs were obtained using the On-Board Imaging System equipped with a linear accelerator. Radiological technologists matched manually the position of the bone structures in the left-right, anteroposterior, and craniocaudal axis using the two orthogonal kV radiographs. After the bone matching, CBCT equipped with a linear accelerator for each fraction was performed with a rotation angle of 204 degrees by splitting the gantry rotation into several BHs.^11,12^ Typically, patients needed three times BHs for CBCT acquisition: each BH was approximately 10 s. After CBCT images were obtained, radiation oncologists and radiological technologists matched manually the position of the daily whole stomach within the PTV. An example of the position matching is shown in Figure 2. After matching, each fraction was delivered.

### Daily target coverage

As a previous study reported daily target coverage using in-room CT,^6^ we assessed daily target coverage of the whole stomach on daily CBCT images (daily CTV). According to the American College of Radiology and the American Society for Radiation Oncology practice parameter for IGRT, an accurate PTV ensures that the prescribed dose is actually delivered to the CTV.^13^ Therefore, we were particularly cautious to deliver the actual doses to the daily CTV as daily target coverage.

Considering interobserver variations in gastric delineation,^14^ daily CTV was delineated individually by two radiation oncologists as observers 1 (ST) and 2 (TN).

Similar to a previous study on daily target coverage using in-room CT,^6^ we also evaluated the coverage of daily PTV that was made by daily CTV plus a 0.8 cm margin which was the same margin used for our radiation treatment planning.

### Statistics

In a previous study on daily target coverage using in-room CT,^6^ we evaluated the V_95%_ (percentage of volume receiving ≥95% of the prescribed dose) of daily CTV and daily PTV using CBCT. Interobserver variations were analyzed using Wilcoxon’s rank-sum test. Statistical significance was defined at *p* < 0.05. We used JMP Pro v. 14 (SAS Institute, Cary, NC) for statistical analyses.

## Results

We assessed six patients who met the inclusion criteria: the median age was 64 years (range, 47–75 years), three patients were male and three female, and the Eastern Cooperative Oncology Group Performance Status of 0.

V_95%_ of the daily CTV for each patient and each observer is listed in Table 1. In total, 102 fractions were assessed; mean V_95%_ of daily CTV in all fractions was 97.2%, which was over 95%. In two of six patients, the V_95%_ of daily CTV was over 95% for either observer in all fractions. One patient had significant interobserver variation in daily CTV (*p* = 0.013); for this patient, the boundary between the stomach and the colon was vague on the daily CBCT images because of gas in both organs.

**Table 1.** V_95%_ of daily CTV and daily PTV for each patient and each observer^a^

	Pt 1		Pt 2		Pt 3		Pt 4		Pt 5		Pt 6	
	Obs 1	Obs 2	Obs 1	Obs 2	Obs 1	Obs 2	Obs 1	Obs 2	Obs 1	Obs 2	Obs 1	Obs 2
Mean V_95%_ of daily CTV (%)	96.3	96.7	96.8	97.1	92.4	97.0	98.1	98.5	99.1	98.7	98.0	98.0
Median V_95%_ of daily CTV (%)	98.7	98.7	97.7	98.3	94.7	97.9	98.5	98.9	99.3	99.1	98.6	98.6
Interquartile range for V_95%_ of daily CTV (%)	95.9–98.9	97.2–98.9	95.7–98.5	97.4–98.6	89.0–97.1	96.8–98.1	97.5–99.1	97.9–99.4	98.7–99.4	98.5–99.3	97.9–99.2	98.3–99.2
Range for V_95%_ of daily CTV (%)	72.6–99.5	77.8–99.4	89.6–99.0	83.6–98.9	80.9–99.4	89.1–99.2	94.0–99.2	95.7–99.6	98.6–99.5	94.2–99.6	92.9–100	93.3–99.4
*p*-value for interobserver variations in daily CTV	0.972		0.428		0.013		0.178		0.312		0.904	
Mean V_95%_ of daily PTV (%)	89.5	91.5	90.8	92.1	82.1	88.8	91.0	92.2	96.4	95.2	90.5	91.5
Median V_95%_ of daily PTV (%)	92.3	95.2	90.1	93.5	81.5	87.4	91.2	93.6	96.6	96.3	91.0	91.8
Interquartile range for V_95%_ of daily PTV (%)	85.3–96.2	88.0–96.8	89.2–94.5	90.0–96.6	77.8–87.1	85.0–94.0	90.0–92.6	90.3–94.2	94.7–97.9	93.7–96.9	89.0–91.7	89.6–93.1
Range for V_95%_ of daily PTV (%)	65.1–98.0	67.1–98.8	80.7–97.0	75.4–97.6	72.4–91.0	80.6–96.1	82.4–94.8	84.1–95.8	92.3–99.0	89.4–99.5	85.6–95.8	86.6–97.1
*p* value for interobserver variations in daily PTV	0.301		0.208		0.003		0.163		0.379		0.202	

CTV, Clinical target volume; Obs, Observer; PTV, Planning target volume; Pt, Patient; V_95%_, Percentage of volume receiving ≥95% of the prescribed dose.

a Considering interobserver variations, daily CTV was delineated individually by two radiation oncologists as observers 1 (ST) and 2 (TN).

In 95 fractions (93%), the V_95%_ of daily CTV was over 95% for either observer. Conversely, V_95%_ of daily CTV was under 95% for both observers in seven fractions (7%). The causes of the seven fractions were as follows: (i) partial dilatation of the stomach by gas (four fractions); (ii) partial positional shift of the stomach by retained stool in the colon (two fractions); and (iii) partial deformation of the stomach (one fraction).

V_95%_ of the daily PTV for each patient and each observer is also listed in Table 1. The mean V_95%_ of daily PTV in all fractions was 91.0%. Significant interobserver variation in daily PTV (*p* = 0.003) was observed in one patient, who was the same patient with significant interobserver variation in daily CTV.

## Discussion

To the best of our knowledge, this is the first study to investigate daily target coverage using CBCT in BH-IGRT for gastric lymphoma, although BH-IGRT is not a new delivery technique and CBCT is a very popular modality in IGRT.^7,8^

It is important to irradiate the target properly; however, data on daily target coverage of radiation are limited in BH-IGRT for gastric lymphoma. A previous study reported good daily target coverage in BH-IGRT for gastric lymphoma.^6^ In this study, daily target coverage was evaluated using in-room CT.^6^ Commonly, in-room CT is not available in every institution, but CBCT is widely used.^7,8^ Therefore, we decided to conduct our study as data regarding daily target coverage using CBCT are needed. A comparison of a previous study using in-room CT^6^ with our study using CBCT is listed in Table 2. Both methods and results seem comparable; mean V_95%_ of daily CTV was 98.5 and 97.2%, respectively, which was over 95% in both studies.

**Table 2.** Comparison of a previous study^6^ with our study

	Previous study^6^	Our study
Institution	Single institution	Single institution
Patients	12 patients	six patients
Fasting	At least 4 h before CT and RT	Abstained from one meal before CT and RT
BH-CT images at planning CT	Three sets	Three sets
BH method	Visual feedback method	Visual feedback method
CTV	Whole stomach	Whole stomach
PTV	CTV plus 0.5–1 cm	CTV plus 0.8 cm
Prescribed dose	30 Gy (1.5–2 Gy/fraction); D_95%_ prescribing method^a^	30.6 Gy (1.8 Gy/fraction); isocenter-prescribing method^b^
Daily CT for BH-IGRT	In-room CT	CBCT
Observer for the study	One observer	Two observers^c^
Mean V_95%_ of daily CTV (%)	98.5	97.2
Mean V_95%_ of daily PTV (%)	94.9	91.0

BH, Breath-hold; BH-CT, Breath-hold computed tomography; BH-IGRT, Breath-hold image-guided radiotherapy; CBCT, Cone-beam computed tomography; CT, Computed tomography; CTV, Clinical target volume; PTV, Planning target volume; RT, Radiotherapy; V_95%_, Percentage of volume receiving ≥95% of the prescribed dose.

*>95%of the PTV was covered by the prescription dose.

a >95%of the PTV was covered by the prescription dose.

b The prescribed dose was delivered to the isocenter.

c Considering interobserver variations, daily CTV was delineated individually by two radiation oncologists as observers 1 (ST) and 2 (TN) in our study.

Although we obtained comparable results for daily CTV, in 7% of all fractions in our study, V_95%_ of daily CTV was under 95% for both observers. The main reasons were: (1) partial dilatation of the stomach by gas and (2) partial positional shift of the stomach by retained stool in the colon. Although we did not use premedication for planning CT and daily BH-IGRT, we should consider using premedication to reduce stomach gas and retained stool for further improvement of daily target coverage for CTV.

Daily PTV was also evaluated in the previous study.^6^ Generally, PTV includes a geometric setup margin and internal target volume (ITV), which is the volume encompassing CTV; this takes into account the fact that CTV varies in position, shape, and size because of physiological factors (respiration, heartbeat, bowel motility, gastric filling).^13^ Although we can minimize the daily setup error and gastric internal motion at the time of CBCT using BH-IGRT, daily intrafractional uncertainties after CBCT and during irradiation remain. To overcome the daily intrafractional uncertainties, daily PTV might also be important. For mean V_95%_ of daily PTV, there seemed to be a difference between the previous study^6^ and our study (94.9 and 91.0%, respectively). We think that this was caused by the difference in dose prescribing methods; in a previous study,^6^ >95% of the PTV was covered by the prescription dose (D_95%_ prescribing method); in our study, the prescribed dose was delivered to the isocenter (isocenter-prescribing method). It is known that dosimetric parameters for PTV such as mean dose, D_95%_, and V_90%_ are higher with the D_95%_ prescribing method than the isocenter-prescribing method.^15^ To further improve the daily target coverage for PTV, we should consider using the D_95%_ prescribing method.

Apart from the stomach, BH-IGRT could be applied for lymphoma of the mediastinum. A study compared no IGRT and BH-IGRT using in-room CT or CBCT for mediastinal lymphoma.^16^ The study showed that although the setup difference between no IGRT and BH-IGRT was measurable, it was not clear whether in-room CT offered an advantage over CBCT in reducing the setup margins: the margin with no IGRT ranged from 7.0 to 17.1 mm, that with in-room CT, from 2.2 to 11.6 mm, and that with CBCT, from 3.6 to 11.1 mm.^16^ The above-discussed comparison between in-room CT^6^ and CBCT for gastric lymphoma seems to be consistent with the results for mediastinal lymphoma.^16^

As for the pros and cons of in-room CT and CBCT, these modalities have the following same advantages: (i) can be used for monitoring patient setup (interfraction motion) and changes in anatomy that have occurred possibly during treatment, by performing imaging immediately after treatment; and (ii) have the ability to monitor tumor response through the course of therapy.^17^ Meanwhile, in-room CT is not available in every institution, but CBCT is widely used.^7,8^ However, CBCT has several disadvantages: (1) suffers from artifacts in the presence of high-density materials (*e.g.* hip prostheses), and (2) patient scatter (especially for larger patients) can degrade image quality.^17^ These cons of CBCT may be a limitation to our study. Considering interobserver variations in gastric delineation,^14^ daily CTV was delineated individually by two observers in our study. As a result, one patient had significant interobserver variation; in the patient, the boundary between the stomach and the colon was vague on the daily CBCT images because of the presence of gas in both organs. It is known that intensified scatter artifacts lower the image quality of CBCT, although CBCT is clinically well-established on IGRT.^18^ The image quality of CBCT may influence interobserver variations by obscuring the organ boundaries. This study had some other limitations: a small number of patients and retrospective single institutional design.

## Conclusion

Daily target coverage for CTV in BH-IGRT for gastric lymphoma seems to be favorable, even when using CBCT.
